# Omega-3 Fatty Acids and PPAR*γ* in Cancer

**DOI:** 10.1155/2008/358052

**Published:** 2008-08-27

**Authors:** Iris J. Edwards, Joseph T. O'Flaherty

**Affiliations:** ^1^Department of Pathology, Wake Forest University School of Medicine, Winston-Salem, NC 27157, USA; ^2^Department of Internal Medicine, Wake Forest University School of Medicine, Winston-Salem, NC 27157, USA

## Abstract

Omega-3 (or n-3) polyunsaturated fatty acids (PUFAs) and their metabolites are natural ligands for peroxisome proliferator receptor activator (PPAR)*γ* and, due to the effects of PPAR*γ* on cell proliferation, survival, and differentiation, are potential anticancer agents. Dietary intake of omega-3 PUFAs has been associated with a reduced risk of certain cancers in human populations and in animal models. In vitro studies have shown that omega-3 PUFAs inhibit cell proliferation and induce apoptosis in cancer cells through various pathways but one of which involves PPAR*γ* activation. The differential activation of PPAR*γ* and PPAR*γ*-regulated genes by specific dietary fatty acids may be central to their distinct roles in cancer. This review summarizes studies relating PUFAs to PPAR*γ* and cancer and offers a new paradigm relating an n-3 PUFA through PPAR*γ* to the expression of the cell surface proteoglycan, syndecan-1, and to the death of cancer cells.

## 1. INTRODUCTION

The peroxisome proliferator-activated receptor (PPAR) family of nuclear receptors comprises three distinct gene products, PPAR*α*, *β*/*δ*, and *γ*, that differ in ligand specificity, tissue distribution, and developmental expression [1–3]. PPARs demonstrate a relatively high level of constitutive transcriptional activity which is further increased upon binding their activating ligands [4–7]. These ligands are primarily long chain unsaturated and polyunsaturated fatty acids (PUFAs) and certain metabolites of these fatty acids [8–10]. Initially, PPARs were thought mainly to govern lipid homeostasis by binding fatty acids and their metabolites to thereby become more active in regulating genes for proteins involved in lipid metabolism [8, 10, 11]. Indeed, PPAR*α* is expressed predominantly in tissues with high fatty acid requirements such as liver, heart, and kidney, while PPAR*γ* isoforms *γ*1 and *γ*2 are highly enriched in adipose tissue to regulate adipocyte differentiation and lipid storage [3]. However, expression of PPAR*γ*1, as with PPAR*β*/*δ* and PPAR*α*, has now been extended to most other tissues and regulatory roles for PPARs extended to other systemic functions such as carbohydrate regulation, immune modulation, and the proliferation, survival and differentiation of cells [3]. The latter effects have led to intense interest in the PPARs in relation to cancer.

PPAR*α* and its ligand activators regulate fatty acid and lipoprotein metabolism and promote the development of hepatocellular carcinoma in rodents and reduce the metastasis of melanoma in hamsters [12]. These and other of their effects do not, in general, translate to humans. PPAR*β*/*δ* plays a key role in lipid metabolism of peripheral tissues. Its high expression in colon has been shown to promote colon cancer [12, 13], in a mechanism that involves the stimulation of PPAR*β*/*δ* by arachidonic acid, PPAR*β*/*δ*-dependent upregulation of cyclooxygenase (COX)-2 leading to overproduction of prostaglandin (PG)E_2_, and PGE_2_-induced growth of colon cancer cells. There is relatively little documentation of a role for PPAR*β*/*δ* in other cancers [14]. By contrast, PPAR*γ* has a broad range of effects on cancer. PPAR*γ* controls fat metabolism by regulating genes involved in lipogenesis, insulin sensitivity, and adipocyte differentiation [3, 15]. These effects underlie the use of thiazolidinediones, which bind and activate PPAR*γ*, to treat insulin-resistant type II diabetes [3, 15]. Although PPAR*γ* activators have been widely shown to inhibit growth in cultured cancer cells, in vivo effects have proved to be complex: they inhibit but sometimes promote cancer growth [16] probably due to stimulation of antiproliferative and apoptotic signaling pathways or proliferative and antiapoptotic pathways, depending on cellular conditions [3, 12, 15–18]. These findings led to the idea of selective PPAR*γ* modulators (SPARMs), drugs analogous to selective estrogen receptor modulators (SERMs) in which distinct actions of the modulator depend on the cellular context [19] and on distinct receptor conformations, and therefore different gene interactions [20]. Fatty acids may be considered as natural SPARMs since their binding does not necessarily lead to PPAR activation and target gene transcription [11].

The considerations discussed above raise a possibility that managed alterations in the type of fatty acids in tissues, can alter the activity of PPARs and thereby the genes they control for therapeutic benefit. The fatty acid content of tissues is dependent mainly on dietary intake. Omega-3 PUFAs, docosahexaenoic acid (DHA), and eicosapentaenoic acid (EPA) are enriched in the diets of many populations that enjoy a low incidence of cancer [21]. These diets also obtain some modest success ameliorating advanced cancer in humans [22] and have been widely used to inhibit carcinogenesis and tumor progression in animal models. The ability of specific fatty acids to differentially activate PPARs and PPAR-regulated genes may be central to their distinct roles in cancer. This review will focus on PPAR*γ*, its activation by fatty acids, and functional results in cancer cells.

## 2. FATTY ACID METABOLISM

### 2.1. Fatty acid types and interconversions

**Figure 1:**
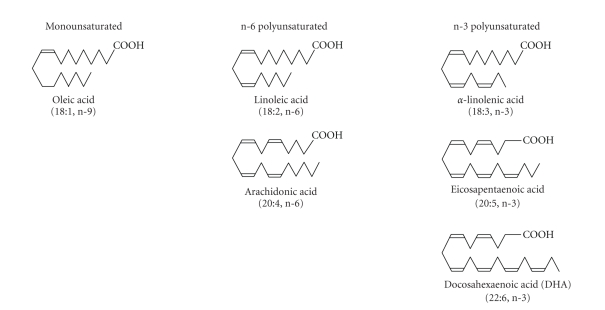
Structures of unsaturated fatty acids: oleic acid (n-9 monounsaturated), linoleic acid and arachidonic acid (n-6 polyunsaturated), *α*-linoleic acid, eicosapentaenoic acid, and docosahexaenoic acid (n-3 polyunsaturated ). The “n” numbers are counted from the methyl or omega terminus.

**Figure 2:**
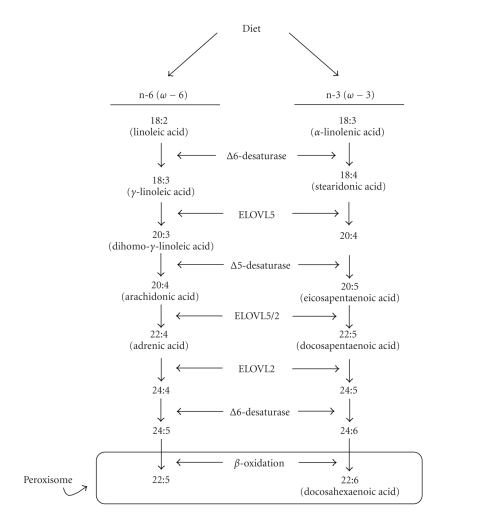
The elongation-desaturation pathway for the metabolism of n-6 and n-3 polyunsaturated fatty acids.

Fatty acids are hydrocarbons with a terminal carboxyl group. The carbons of saturated fatty acids are all connected by single bonds, whereas the chains of monounsaturated and polyunsaturated fatty acids (PUFAs) contain one or more double bonds, respectively. The n-3 and n-6 designation describes the position of the double bond closest to the (omega) carbon at the methyl end of the molecule (Figure 1). Oleic acid (18 : 1) has a single double bond between carbons 9 and 10 from the omega carbon and is designated an n-9 or omega-9 monounsaturated fatty acid. Like the saturated fatty acids, oleic acid can be synthesized de novo in mammalian cells. It can also be obtained from the diet through intake of oils such as olive and canola. By contrast, PUFAs cannot be synthesized de novo in mammals and must be obtained from the diet. The shortest of the n-6 PUFAs is linoleic acid (LA, 18 : 2, n-6). Its 18 carbon, n-3 counterpart is *α*-linolenic acid (ALA, 18 : 3, n-3). Both LA and ALA are metabolized through a series of elongation and desaturation steps to longer chain PUFAs: LA to arachidonic acid (AA, 20 : 4, n-6) and ALA to EPA (20 : 5, n-3) and DHA (22 : 6, n-3) (Figure 2). The first and rate limiting step in this pathway is the introduction of a double bond by the Δ6 desaturase (for review see [23]). For n-3 PUFAs, ALA is converted to stearidonic acid (SDA, 18 : 4, n-3), elongated, and desaturated by Δ5-desaturase to form EPA. In mammalian cells, the conversion of EPA to DHA follows the Sprecher pathway in which EPA is elongated to docosapentaenoic acid (DPA, 22 : 5, n-3), then to tetracosapentaenoic acid (TPA. 24 : 5, n-3), and desaturated to tetracosahexaenoic acid (THA, 24 : 6, n-3). THA is translocated from the endoplasmic reticulum to peroxisomes, where *β*-oxidation results in the loss of 2 carbons to form DHA [24]. The PUFAs are also metabolized, most importantly for this review, to PPAR*γ* activators (see Section 2.3).

### 2.2. Dietary fatty acids

The results of both dietary intake and stable isotope studies have shown that the conversion of ALA to DHA in humans is extremely inefficient (for review see [25]). Most of the ingested ALA is an immediate target for *β*-oxidation to provide energy, leaving an estimated 8–10% to enter the elongation-desaturation pathway [26, 27]. A kinetic analysis of ^2^H-labeled fatty acids estimated that conversion of ALA to EPA was only 0.2%, EPA to DPA was 0.13%, and DPA to DHA was 0.05% [28]. There is some evidence of gender-related differences in the activity of the elongation-desaturation pathway that result in a greater efficiency of conversion of ALA to DHA in females than in males [25, 27, 29]. Support for a role of sex hormones in the conversion pathway is provided by data indicating higher DHA in plasma lipids associated with oral contraceptive use [27] as well in males supplemented with estrogen during sex-change procedures [30]. Moreover, testosterone treatment of female-male transsexuals was shown to decrease plasma DHA [30].

Because common enzymes in the elongation-desaturation pathway are responsible for conversion of both n-3 and n-6 PUFAs, background diet is also a factor in efficiency of conversion. LA is the most abundant fatty acid in the Western diet with consumption in US that is ten-fold that of ALA (reviewed in [31]). Studies have shown that a high intake of LA is associated with a low conversion of ALA to EPA [26]. In spite of limited metabolism of ALA to its long chain derivatives in the stable isotope tracer studies, feeding studies have consistently shown that increased consumption of ALA does result in higher levels of EPA in plasma or cell lipids [31]. However, there was no measurable increase in DHA in these pools. Likewise diets supplemented with EPA do not result in a detectable increase in plasma DHA [32]. Thus, the inefficiency of this pathway does not appear limited to one step but rather extends throughout the pathway. The consensus of a number of studies is that the only way to increase plasma and tissue levels of a specific PUFA is to increase the consumption of that fatty acid. This may be of particular importance in the light of recent in vitro studies on the antitumor effects of DHA.

### 2.3. PUFAs metabolism to PPAR*γ* activators

**Figure 3:**
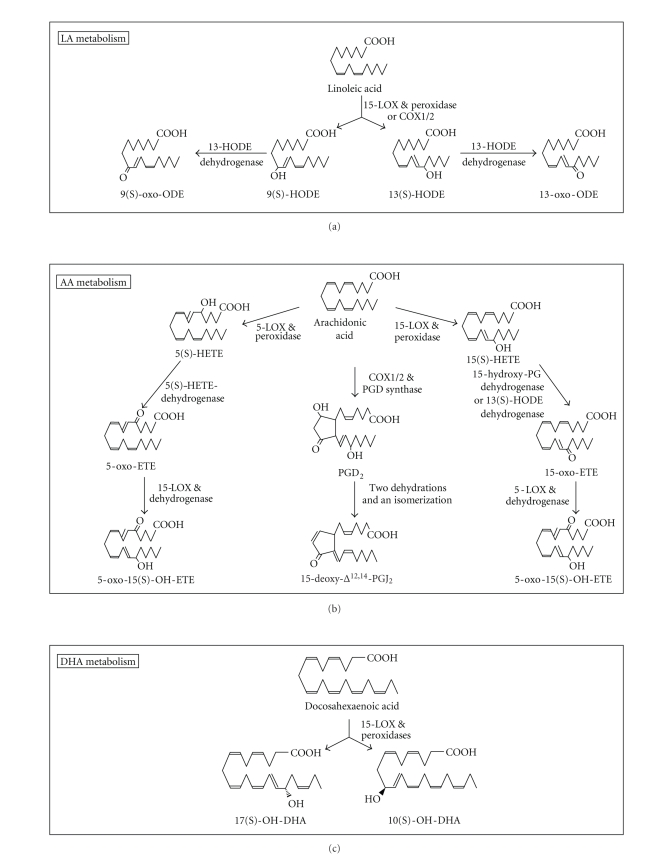
The cellular metabolism of LA, AA, and DHA to more potent activators of PPAR*γ*. ODE is octadecaenoate; HETE is hydroxy-eicosatetraenoate; ETE is eicosatetraenoate; PG is prostaglandin.

Tissues metabolize PUFAs to oxygenated products that have quite different impacts on PPAR*γ* than their parent molecules. Moreover, n-3 PUFA inhibit the metabolism n-6 PUFAs and subplant them from their oxygenation pathways to form products [33–35]that have different effects on PPAR*γ* than their n-6 PUFAs oxygenated counterparts. It is therefore important to consider PUFAs oxygenation pathways. LA, AA, and DHA require >10–30 *μ*M to activate PPAR*γ* but are commonly converted to stronger (>0.1–10 *μ*M) activators in cells. LA is metabolized (Figure 3, upper panel) by 15-lipoxygenases (LOX)-1/2 to 9(S)- and 13(S)-HODE (hydroxy-octadecaenoate) and by cyclooxygenases (COX)-1/2 to 9(R)- and 13(S)-HODE. The HODEs can be converted to 13-oxo- and 9-oxo-ODE by a dehydrogenase [36–39]. The hydroxy and to a greater extent oxo LA analogs have greater PPAR*γ*-activating potency than LA [36, 40–42]. AA is metabolized (Figure 3, center panel) via 5-LOX to 5(S)-HETE (hydroxy-eicosatetra-enoate) and via 15-LOX-1/2 to 15(S)-HETE. These HETEs can be converted to oxo-ETEs and 5-oxo-15(S)-hydroxy-ETE as shown in Figure 3 [39, 43–50]. 15-HETE has weak and 5-HETE essentially no ability to activate PPAR*γ*. However, their oxo counterparts have appreciable ability to do so with 5-oxo-15(S)-hydroxy-ETE showing the greatest potency in binding and activating PPAR*γ* [43]. AA is also metabolized (Figure 3, center panel) by COX1/2 to PG (prostaglandin) D_2_ which as a consequence of successive dehydrations and an isomerization, perhaps by nonenzymatic routes, convert to PGJ_2_, Δ^12^-PGJ_2_, and 15-deoxyΔ^12,14^-PGJ_2_(15-d-Δ^12,14^-PGJ_2_); these PGJ_2_'s have greater ability than PGD_2_ to activate PPAR*γ* with 15-d-Δ^12,14^-PGJ_2_ being a most potent (>0.1–1 *μ*M) naturally occurring PPAR*γ* activator [9, 43, 51–56]. In one study, the *K_d_*'s of 15-d-Δ^12,14^-PGJ_2_, 5-oxo-15-OH-ETE, PGJ_2_, 5-oxo-ETE, and 5(S)-HETE in binding to PPAR*γ* were 1.4, 11, 37, 81, and >1000 *μ*M, respectively; their potency in activating a cell-based PPAR*γ* reporter paralleled these *K_d_*'s [43]. DHA is metabolized (Figure 3, bottom panel) by 15-LOX or other oxygenase to 17-OH- and 7-OH-DHA, products that activate PPAR*γ* with greater potency (ED_50_'s in activating a cell-based PPAR*γ* reporter of ~5 *μ*M) than DHA [57]. 4-OH-, and 4-oxo-DHA [53], while not yet shown to be made by cancer cells, also activate PPAR*γ* with greater potency (ED_50_'s of 13.4 and 7.8 *μ*M in activating a cellular PPAR*γ* reporter, resp.) than DHA (ED_50_ > 10 *μ*m) [53]. Hence, in this DHA series, similar to the 5-HETE series of AA metabolites, the oxo analog exhibits the greatest potency. We note that the more potent PPAR*γ* activators, the oxo-PUFAs, form preferentially in cells undergoing excessive oxidation, free radical, and NADPH/NADH-depleting reactions [43, 44, 48, 57, 58]. This suggests that PPAR*γ* may serve as a sensor for oxo-PUFA thereby monitoring cellular oxidative stress and when this stress is severe, engaging cell death programs [43, 58]. This PPAR*γ* function, we suggest, could contribute to the necrosis that occurs in tumors particularly after chemical and radiation treatment [59].

Cells process PUFAs in other relevant ways. They convert them to nitrates, probably in nonenzymatic reactions, where the nitric oxide made during cell stimulation attacks the PUFAs. Nitrated LA and AA are stronger PPAR*γ* activators than their precursors [60–62]. Cells also convert PUFAs to cannabinoids such as anandamide (ethanolamine amide of AA) and arachidonoylglycerol which also activate PPAR*γ* with greater potency than AA [63–65]. Finally, cells conjugate glutathione to PUFAs that contain an *α*,*β*-unsaturated ketone such as 15-d-Δ^12,14^-PGJ_2_ and 5-oxo-ETE [66–68]. Since the conjugates are rapidly excreted from cells by multidrug-resistance transporters, conjugation inhibits the ability of *α*,*β*-unsaturated ketones to activate PPAR*γ* [66]. Cancer cells excrete anticancer drugs through these same transporters and become drug-resistant by overexpressing these transporters [69]. Such mutated cells may also be resistant to *α*,*β*-unsaturated ketone activators of PPAR*γ*.

### 2.4. Low-density lipoproteins (LDL) as deliverers of PPAR*γ*-activating n-3 PUFA

LDL carry esterified PUFAs in glycerolipids and cholesterol. They bind to cell surface LDL receptors and then internalize in endocytic vesicles which merge with lysosomes to de-esterify and release the PUFAs into the cytosol [70]. This route differs from the direct delivery of PUFA: it bypasses cell surface G protein-coupled fatty acid receptors (GPR 40 and 120; see Section 4.3), deposits PUFA in cells more slowly, and thereby avoids stimulation of G protein-coupled receptors and, perhaps, an array of C domain-bearing proteins which are activated by PUFA. This is also an important pathway for delivering PUFA to tumor cells because of a significant increase in LDL receptor activity in neoplastic tissues [71–73]. We have obtained from monkeys fed special diets, LDL enriched with n-6 PUFA (mostly AA and LA) or n-3 PUFA (mostly DHA and EPA). The n-3 but not n-6 PUFA-rich LDL mimicked thiazolidinediones and DHA in inhibiting cancer cell growth [74] and activating PPAR*γ* [75, 76].

## 3. PPAR*γ*

### 3.1. Structural considerations

PPAR*γ*1 and *γ*2 originate from the PPAR*γ* gene through separate promoters and 5′ exons. Compared to the ubiquitously expressed PPAR*γ*1, PPAR*γ*2, which is limited mainly to adipose tissue, has 30 additional amino acids at its NH^2^ terminus and is a more potent transcription activator [77]. Because they appear to have the same targets, however, the two isoforms are here considered together under the term PPAR*γ*. PPAR*γ* is comprised of four functional parts: the NH_2_-terminal A/B region bears a ligand-independent transcription-activating motif AF-1; C region binds response elements (PPREs with a DR-1 consensus half-sequence of AGGTCA); D region binds various transcription cofactors; and E/F region has an interface for dimerizing with 9-*cis* retinoic acid receptors (RXRs), an AF-2 ligand-dependent transcription-activating motif, and a ligand-binding domain (LBD) [3, 12, 15, 17]. The LBD has a spacious cavity that binds ligands having a polar head group extending from a hydrophobic tail such as diverse PUFAs and PUFA metabolites [7, 77].

### 3.2. PPAR*γ* regulation by other signaling pathways

PPAR*γ* is phosphorylated by extracellular signal-regulated kinases (ERK)-1/2 and C-Jun N-terminal kinase; when so phosphorylated, it has less ligand-binding affinity and gene-regulating activity [3, 78, 79]. The phosphorylation and attendant decrease in activity of PPAR*γ* occur in cells treated with PPAR*γ* activators and may cause the activators to show little or no ability to stimulate PPAR*γ* [3, 79–81]. ERK pathways impact PPAR*γ* in another way: the ERK-activating enzyme, MEK, when activated, binds with PPAR*γ*'s AF-2 motif. This causes PPAR*γ* to release from PPRE complexes and, bound to MEK and directed by MEK's nuclear export signal, to exit the nucleus [81, 82]. It is important to note that PUFAs and PUFA metabolites can activate the MEK/ERK pathway (see Section 4.3) and therefore may have biphasic effects: they not only directly activate PPAR*γ* but also entrain events inhibiting PPAR*γ*.

PPAR*γ* is targeted for degradation by ubiquitylation and sumoylation. Ligand binding, certain protein kinases, and some transcription cofactors (e.g., p300) promote ubiquitin-dependent degradation of PPAR*γ* in proteasomes [3]. Sumoylation occurs on K107 of PPAR*γ*2 in a ligand-independent fashion to inhibit AF-1 function and on K365 of PPAR*γ* in a ligand-dependent fashion to promote PPAR*γ*'s binding of nuclear receptor corepressor [83, 84]. Sumoylation of PPAR*γ* causes its proteasomal degradation. ERK phosphorylation promotes K107 sumoylation. This reaction represents yet another means by which ERKs can inhibit PPAR*γ* [84].

### 3.3. PPAR*γ* transcriptional cofactors

PPARs bind a specific DNA sequence termed peroxisome proliferator response element (PPRE) in the 5′-flanking region of target genes as a heterodimer with RXR. Studies using various techniques [3, 85, 86] suggest the following model: PPAR*γ*•RXR complexes (the interaction is ligand-independent) exist in nuclei as macrocomplexes associated with various transcription corepressors [3, 87]. Some complexes, ligand-bound or not, may associate with transcription coactivators to control the basal expression of genes. In any event, PPAR*γ*•RXR complexes are highly mobile, rapidly scanning chromatin, although this scanning does not involve their DNA binding domain [86]. Ligands trigger PPAR*γ*•RXR to localize at their cognate PPREs and to exchange corepressors for coactivators such as cyclic AMP response element binding protein (CREB) and p300 [3, 16, 87, 88]. At some gene sites, activators cause PPAR*γ*•RXR to recruit corepressors and thereby cause gene repression [3, 89, 90]. However, the availability of cofactors differs between cell types and within cells over time depending on the cell's history and the association of the cofactors to other genes [3, 15, 16], for example, activation of PPAR*γ* deprives T cell factor/lymphoid enhancing factor (TCF/LEF) of cofactors to thereby inhibit oncogenic signaling by the Wnt pathway [16]. Thus, the effects of PPAR*γ* activation vary depending on context and cofactor availability at each genetic site. It seems at least possible that the PUFA ligands for PPAR*γ* will have differential effects in impacting its interactions with these transcriptional cofactors in a manner similar to the SPARMs model [19].

## 4. TARGETS OF PPAR*γ* RELEVANT TO CANCER

### 4.1. Gene targets of PPAR*γ*

Most known target genes of PPAR*γ* regulate lipid metabolism and transport [15]with few cancer-related genes having been confirmed as induced by PPAR*γ*. PPAR*γ* does induce G_0_/G_1_ switch gene 2 whose product causes growth arrest in 3T3-L1 cells [91, 92]. PPAR*γ* also binds the NF*κ*B promoter of p53 to stimulate expression of p53 and, in consequence, p21^WAF1/Cip1^. It also binds to a promoter in the Fas ligand gene to induce the expression of this member of the extrinsic apoptosis pathway. These effects appear responsible for slowing growth and causing apoptosis in MCF7 breast cancer [93], human umbilical vein endothelial [94], and possibly Reh [95] cells. Recent studies have identified the heparan sulfate proteoglycan, syndecan 1, as a target for PPAR*γ* in human breast [75, 76] and prostate [96] cancer cells. The upregulation of syndecan 1 by PPAR*γ* resulted in apoptosis induction [76].

### 4.2. Other targets of PPAR*γ*

PPAR*γ* impacts many growth-promoting elements through its secondary actions that, while ligand-dependent, do not directly involve its gene promoters. It interacts with nuclear factor of activated T cells, phosphorylated signal transducer, and activator of transcription (STAT)-3, and nuclear factor *κ*B (NF*κ*B) to block signaling through these pathways [3]. It binds transcription cofactors to alter these cofactors' availability to other transcription factors: ligand bound-PPAR*γ* deprives NF*κ*B of AP-1; deprives STAT-1 of CREB binding protein; and releases SMRT to render it available to repress STAT-3's transcriptional activity [3, 16, 17, 97]. PPAR*γ* activation is also associated with the activation of ERK1/2, protein kinases C, protein kinase A, AMP-activated protein kinase *α* [17]; induction of p16, p18, and p21 cyclin-dependent kinase inhibitors [3, 17, 18]; decreased expression of cyclooxygenase 2, *cmyc*, *cmyb*, D1, and D3 cell cycle control genes, and regenerating gene 1A [17, 18]; decreased secretion of cytokines and growth factors [17, 98]; depression of the Akt survival pathway by upregulating PTEN and inhibiting the phosphorylation of Akt and mTOR [3, 17]; inhibiting retinoblastoma protein (Rb) activity to repress the activities of cyclins D3 and E [3]; and regulating a host of other elements involved in the growth and death of cells [3, 12, 16–18]. It is not clear which if any of these effects are due to the action of PPAR*γ* or PPAR*γ* activators. PUFAs impact many of these same targets but can do so not only by PPAR*γ*-dependent but also PPAR*γ*-independent routes (see the next section).

### 4.3. Targets of PPAR*γ*-activating Ligands

Studies of PPAR*γ* function depend on challenging cells with PPAR-activating ligands that have numerous side effects impacting cell growth. 15-d-Δ^12,14^-PGJ_2_ has a reactive *α*,*β*-unsaturated ketone (Figure 3) that covalently binds to cysteine sulfur on PPAR*γ*; this renders its PPAR*γ* binding irreversible [58, 68]. 15-d-Δ^1,14^-d-PGJ_2_ also binds to cysteines in the IKK*β* subunit of I*κ*B kinase, thereby inhibiting NF*κ*B activation [99, 100]. Other ligands with an *α*,*β* unsaturated ketone (e.g., oxo-ODEs and oxo-ETEs; see Figure 3) have this chemical reactivity [58] and along with 15-d-Δ^1,14^-d-PGJ_2_ may exert anticancer effects by covalently attaching to signal molecules like IKK*β* [58, 99, 101] or elements needed for expressing the epidermal growth factor receptor (EGFR) and JAK [102, 103].

Naturally occurring ligands have other PPAR*γ*-independent effects. The D and J series of PGs including 15-d-Δ^12,14^-PGJ_2_ bind to PGD_2_ receptors [104], 5-oxo- and 5-oxo-15-hydroxy-ETE bind to the OXE receptor [105], and AA, EPA, and DHA bind to GPR40 and GPR120 receptors [106, 107]. These G protein-coupled receptors regulate signal pathways that effect cancer cell growth. For example, 5-oxo-15-hydroxy ETE acts on OXE to stimulate cells to activate ERK and Akt and proliferate; this stimulation counters its antigrowth activity in various cancer cell types. Indeed, HEK293 cells lack OXE receptors and in contrast to OXE receptor-bearing breast, prostate and ovarian cancer cell lines respond to 5-oxo-ETE and 5-oxo-15-oxo-ETE only by slowing, not speeding, their proliferation [43]. PUFAs activation of GPR120 also causes ERK and Akt activation to increase the survival of serum-starved STC-1 cells [108]. Finally, PUFAs are also metabolized to products that act on G protein receptors to promote cell growth, for example, prostate cancer cells convert AA to PGE_2_, which acts through its receptors to stimulate the NF*κ*B pathway and thereby the expression of various cytokines and growth factors [109]. The G protein receptor-dependent actions of PPAR*γ* ligands may explain reports that these ligands have biphasic effects in stimulating proliferation and antiproliferation in cancer cells [110].

Thiazolidinediones stimulate cells to activate ERK1/2, p38, and JNK [111–113] by discharging Ca^2+^ from the ER to evoke an ER stress response; this activates Ca^2+^/calmodulin kinase II, proline-rich tyrosine kinase 2, protein kinases C, *c-Src*, EGFR, the ERK1/2 and JNK pathways, the double stranded RNA-activated protein kinase, and p38 [111]. Double stranded RNA-activated protein kinase inactivates eukaryotic initiation factor-2 to depress protein translation [111, 114]. Since EPA has recently been shown to have similar effects on ER calcium discharge [111, 115], it seems likely that various other PUFAs activate the ER stress pathway. Nonetheless, PPAR*γ* activators often show very different side effects [42, 103, 116–120]. For example, among three PPAR*γ* agonists, ciglitazone, 9-HODE, and 13-HODE, only 9-HODE induced apoptosis in U937 cells [38], 15d-Δ^12,14^-PGJ_2_, but not various other PPAR*γ* ligands, reduced EGFR expression in squamous carcinoma cells [99], 15d-Δ^12,14^-PGJ_2_, but not troglitazone, inhibited the stimulated induction of MHC class II molecules in retinal pigmented epithelial cells [112], and DHA, but not EPA, stimulated the target gene, syndecan 1 to inhibit the proliferation and induce apoptosis in breast and prostate cancer cell lines [75, 76, 96]. Numerous other examples of differential effects among PPAR*γ* agonists exist (e.g., [113–116]), but it is worth stressing that n-3 PUFAs inhibit the metabolism of n-6 PUFAs to products that promote the growth of cancer cells such as PGE_2_, 5-HETE, and leukotriene B_4_ [33–35, 45, 113]. This inhibitory effect may make an important contribution to the anticancer effects of n-3 PUFAs.

## 5. DIETARY FATTY ACIDS AND CANCER

### 5.1. Human studies

Although there are inconsistencies [121], human population studies have shown that consumption of a diet enriched in n-3 PUFAs may offer protection against a number of cancers including those of breast [122–124], prostate [125, 126], and colon [127–129]. Although many of these studies have relied on dietary intake data from self-reported questionnaires or estimates based on national consumption, a few have used the fatty acid composition of tissues as a measure of exposure to dietary fats. The EURAMIC study is one of the largest to provide evidence that the balance between n-3 and n-6 PUFA may play a role in breast cancer [130]. Adipose tissue aspirates from breast cancer patients and controls demonstrated that the ratio of long chain n-3 to n-6 PUFAs was inversely associated with breast cancer in four of five centers studied. In human prostate tissue, lower EPA and DHA as well as lower n-3 to n-6 PUFAs ratios were associated with cancer compared to benign prostate hyperplasia [131] and with advanced stage compared to organ confined disease [132]. This inverse association of n-3 PUFAs and prostate cancer is supported by analyses of fatty acids in serum and red-cell membranes of patients with prostate disease [133, 134].

### 5.2. Animal studies

Animal studies provide convincing evidence of a negative relationship with n-3 PUFA diets and a positive relationship with n-6 PUFA diets for breast, prostate, and colon cancer. In studies of breast cancer induced by chemical carcinogens in rats [135–137], and human cancer cell xenografts in nude mice [138–140], tumor growth rate, size, and metastases were all suppressed by dietary n-3 PUFA supplementation. Likewise for colon cancer, antitumor properties of n-3 PUFA diets have been shown in transplantable mouse tumors [141–143] as well as in chemically induced rat tumors [144–151]. Although there have been fewer animal studies of PUFAs in prostate cancer, they are consistent with those in breast and colon cancer. In xenograft models of prostate cancer, n-3 PUFAs enriched diets inhibited tumor growth compared to n-6 PUFA diets [152–154]. Recently, a prostate-specific Pten knockout mouse model was used to demonstrate that a dietary ratio of n-6 to n-3 PUFA lower than 5 was effective in suppressing tumor growth, and extending animal lifespan [155].

### 5.3. Cell culture studies

Insight into the mechanism(s) responsible for the anticancer properties of n-3 PUFAs have been provided by animal studies as well as by in vitro investigations using human cancer cell lines. A major focus for such studies has been the competitive inhibition between n-6 and n-3 PUFAs for the enzymes involved in their metabolism. The desaturation and elongation of LA to AA were shown to be decreased in the presence of high n-3 PUFAs due to enzyme preference for the n-3 substrates [156]. AA and EPA compete for the COX and LOX enzymes, again with preferential n-3 utilization that results in a reduction in the highly reactive eicosanoids generated from AA [157, 158] in favor of less inflammatory n-3 eicosanoids [159]. The decreased growth of prostate xenograft tumors was shown to involve inhibition of COX 2 and PGE_2_ in the tissues [154]. Thus, the combined human, animal, and cell culture studies indicate that diet is an important regulator of the levels of n-3 versus n-6 PUFAs in tissues, including those that are cancerous. High levels of n-3 PUFAs may directly evoke antitumor events, become metabolized to products with antitumor activity, or suppress the production of tumor-promoting metabolites such as those formed by n-6 PUFAs.

## 6. n-3 PUFA REGULATION OF SYNDECAN-1

**Figure 4:**
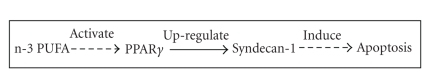
The syndecan-1 pathway for n-3 PUFA induction of apoptosis. Dashed lines indicate that effects may be indirect with involvement of other metabolites and signaling molecules.

Increasing evidence implicates PPAR*γ* in the divergent effects of n-3 and n-6 PUFAs in cancer cells and point to a growth inhibitory role for PPAR*γ* [160–164]. We recently found that n-3 PUFAs—but not n-6 PUFAs—enriched LDL, inhibited the proliferation, and induced apoptosis in human breast cancer cells [74–76]. The n-3 LDL delivered both EPA and DHA to the cells. When these individual fatty acids were delivered to cells by albumin, DHA but not EPA proved effective in stimulating apoptosis in a pathway that involved activation of PPAR*γ* [75]. The molecular target for both DHA and PPAR*γ* in these cells was shown to be the heparan sulfate proteoglycan, syndecan-1. Syndecan-1 itself was effective in apoptosis induction and when syndecan-1 was silenced, the ability of DHA to induce apoptosis was completely blocked as it was in the presence of a dominant negative PPAR*γ* [76]. Moreover, syndecan-1 siRNA was effective in blocking troglitizone-induced apoptosis. Thus, a novel pathway linking n-3 PUFAs to apoptosis in tumor cells is as follows: DHA activates PPAR*γ*, which results in transcriptional upregulation of the syndecan-1 target gene, and the syndecan-1 protein induces apoptosis (Figure 4). This novel pathway has been confirmed in human prostate cancer cells [96].

Although PPAR*γ* was not a target for EPA in breast and prostate cancer cells, a recent report has demonstrated that EPA was an effective PPAR*γ* transactivator in HT-29 human colon cancer cells [165]. In contrast, both EPA and DHA were shown to reduce PPRE reporter activity in an HCT-116 colon cancer cells [166]. DHA has recently been shown to reduce the growth of human lung cancer cells in a process that was associated with increased PPAR*γ* protein [167]. These conflicting reports are consistent with data showing selective modulation of PPAR*γ* by different ligands in different cells [168]. Several other reasons may be proposed for the differential response to DHA and EPA in the breast and prostate tumor cells including (1) PPAR*γ* activation may be mediated by a unique DHA metabolite rather than DHA itself; (2) there may be a difference in the bioavailability of the two fatty acids following cellular uptake; (3) EPA may be a ligand for or metabolized to a ligand (e.g., 5(S)-hydroxy-eicosapentaenoic acid) for a G protein-coupled receptor that activates ERK and thereby inactivates or in some other way counteracts PPAR*γ*; (4) EPA may directly, or after being metabolized, activate other pathways that counteract PPAR*γ* signaling.

The identification of syndecan-1 as a target gene for PPAR*γ* in the breast and prostate cancer cells was a novel but not unexpected finding. The syndecan-1 promoter contains a DR-1 element that is recognized by a several members of the nuclear hormone receptor superfamily including PPAR*γ*. Although there are conflicting reports of a role for syndecan-1 in cancer, the importance of these studies is the identification of a PPAR*γ* molecular target that is regulated by PUFAs and results in functional response in the tumor cells. As more such targets emerge, we may be able to understand how different dietary fatty acids play divergent roles in cancer.
